# *QuickStats:* Percentage* of Adults Aged 50–75 Years Who Received Colorectal Cancer Screening,^†^ by Poverty Status^§^ and Year — National Health Interview Survey, United States, 2010 and 2018^¶^

**DOI:** 10.15585/mmwr.mm6929a6

**Published:** 2020-07-24

**Figure:**
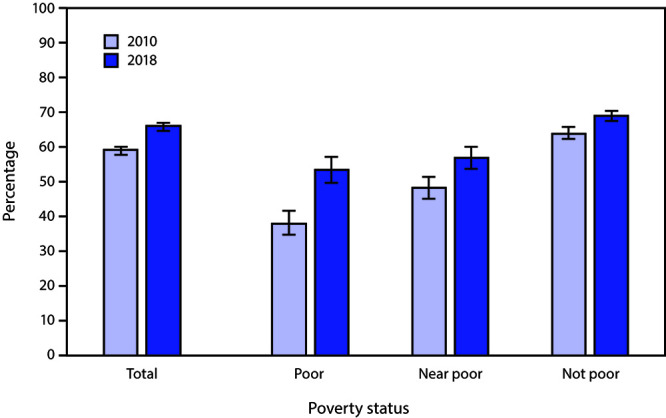
The percentage of adults aged 50–75 years who received colorectal cancer tests or procedures increased from 58.7% in 2010 to 65.5% in 2018. The percentage increased from 2010 to 2018 in all income groups: from 37.9% to 53.1% among poor, 47.9% to 56.7% among near poor, and 63.6% to 68.7% among not poor adults. In both 2010 and 2018, the percentage of adults who received colorectal cancer screening was lowest among poor and highest among not poor adults.

**Source:** National Health Interview Survey, 2010 and 2018. https://www.cdc.gov/nchs/nhis.htm.

